# Prevalence and related factors of depressive symptoms in hemodialysis patients in northern China

**DOI:** 10.1186/s12888-017-1294-2

**Published:** 2017-04-05

**Authors:** Xiaodan Liu, Xiaoshi Yang, Li Yao, Quan Zhang, Da Sun, Xinwang Zhu, Tianhua Xu, Qiang Liu, Lining Wang

**Affiliations:** 1 Department of Nephrology, The First Hospital of China Medical University, No.155 North Nanjing Street, Shenyang, Liaoning, People’s Republic of China 110001; 2 Department of Social Medicine, School of Public Health, China Medical University, No.77 Puhe Road, Shenyang North New Area, Shenyang, Liaoning, People’s Republic of China 110013; 3 Department of Nephrology, General Hospital of Fushun Mining Bureau, No.24, Zhongyang Ave., Xinfu Dist. Fushun, Liaoning, People’s Republic of China 113008

**Keywords:** Depressive symptoms, Hemodialysis patients, Activities of daily living, Social support, Coping style, Ego resiliency

## Abstract

**Background:**

To determine the prevalence of depressive symptoms and to explore related factors of depressive symptoms in hemodialysis (HD) patients in northern China.

**Methods:**

We used a cross-sectional research design to recruit 227 chronic kidney disease (CKD) patients who were undergoing HD treatment in northern China during December, 2012 to March, 2013. The Chinese edition of the Center for Epidemiologic Studies Depression (CES-D) was used to measure depressive symptoms. Information on quality of life (QOL), activities of daily living (ADL), social support status, coping style, self-efficacy, ego resiliency and demographic characteristics was all collected by face to face interview. Multivariate logistic regression analysis was used to explore related factors of depressive symptoms.

**Results:**

The prevalence of depressive symptoms among HD patients was 29.1%. Patients with a lower mood have worse ADL and QOL than patients with better mood. Patients with a lower mood have got less social support than patients with better mood, including both family support and outside family support. For coping style, patients with a lower mood were more inclined to choose “acceptance-resignation” coping style than patients with better mood, while the result is opposite in “avoidance” coping style. And patients with a better mood have better self-efficacy and ego resiliency than patients with lower mood. Multivariate logistic regression analyses revealed that ADL (OR = 1.124, *p* = 0.002), family support (OR = 0.867, *p* = 0.021), “acceptance-resignation” coping style (OR = 1.228, *p* = 0.022), and ego resiliency (OR = 0.944, *p* = 0.021) were associated with low mood independently.

**Conclusions:**

The prevalence of depressive symptoms is high in CKD patients on HD in northern China. activities of daily living, family support, “acceptance-resignation” coping style and ego resiliency were independently associated with depressive symptoms.

## Background

With the prevalence of chronic kidney disease (CKD) at 10.8%, there are up to 119.5 million patients with CKD in China. A large portion of patients with CKD progress to end-stage renal disease (ESRD), which need renal replacement therapy [1, 2]. At the end of 2013, the number of patients receiving dialysis was 326,000 in mainland China, which puts a huge burden on patients and the society [3–5].

Depression is a chronic and recurrent illness. Patients with CKD on hemodialysis (HD) have to endure physical discomforts related to their illness and have to face various stresses, family problems, which increasing the risk of depression [6]. The prevalence of depression is higher among patients receiving HD, with estimated rates ranging from 23 to 42% in the USA and Europe, and 45.9% in Taiwan [7–11]. Furthermore, clinical depression and subthreshold depressive symptoms are strongly associated with poor adherence to treatment, increased mortality and rates of hospitalization [12–16].

Although an association between depression and HD has been strongly suggested, the prevalence and related factors of depressive symptoms among CKD patients on HD in northern China are largely unknown. The main purposes of this study were to (1) assess the prevalence of depressive symptoms of HD patients in northern China, (2) explore demographic and clinical characteristics that might identify patients at high risk for depressive symptoms, (3) make comprehensive assessment by applying questionnaires of quality of life (QOL), social support, coping style, self-efficacy and ego resiliency, and try to find correlates of depressive symptoms of patients on HD.

## Methods

A cross-sectional study was conducted by cluster sampling in two affiliated hospitals of China Medical University, from Dec 12, 2012 to Mar 15, 2013. Patients with CKD who received HD for at least one month participated in this study, and were face to face interviewed. Patients were interviewed while receiving dialysis treatments by medical students who were extensively and repeatedly trained by psychiatry faculty. All of the participants were well informed and had written informed consent. The study was approved by the Ethics Committee on Human Experimentation of The First Hospital of China Medical University.

The inclusion criteria for study participation were as follows: (1) age > 16 years; (2) ESRD patients who were undergoing HD therapy for at least one month; (3) had no history of transplant. The exclusion criteria were (1) younger than 16 years old; (2) patients with dementia or cognitive impairment which were defined by self or family report; (3) patients with unconsciousness or inability to read or write; (4) patients with severe complications of the heart, lung, brain and other parts of the body; (5) previous treatment for depression. There was no significant difference in age, sex and disease between excluded patients with depression and selected patients.

### Demographic and clinical characteristics

Demographic characteristics included age, gender, marital status (married/others), educational level (≤junior high school/> junior high school), financial status, HD duration and patient’s functional status.

Data related to clinical characteristics, including cause of CKD, duration of renal disease, and ionic concentration were collected from hospital records. In addition, concern about arteriovenous fistula was also collected from patient interviews.

### Questionnaires

#### Measurement of depressive symptoms

Chinese edition of the Center for Epidemiologic Studies Depression (CES-D) was used to measure depressive symptoms [17]. It includes 20 items, with each one comprising four categories: (0) never, (1) sometimes, (2) frequently and (3) always. Scores of 16 or more are defined as depressive symptomes. Participants were divided into groups of low and normal mood by score of 16. It has been widely used in Chinese sample with satisfied validity and reliability (Cronbach’s alpha was 0.8895).

#### Measurement of functional status

Activities of Daily Living Scale (ADL) was used to measure patient’s functional status [18]. ADL, a 14-item self-rating scale can be categorized into basic ADL and instrumental ADL, which evaluated by six items (i.e. bathing, dressing, walking, toileting, tooth brushing and eating) and eight items (i.e. meal preparation, shopping, clothes washing, telephone using, finances managing, transferring, doing housework, and medicine taking) respectively [19]. Each item is scored on a 4-point scale, and higher score indicates a higher level of dependence. (Cronbach’s alpha was 0.8709).

#### Assessment of quality of life

QOL was evaluated by using the 36-item Short-Form Health Survey (SF-36). The SF-36 measures eight different dimensions of health, which is described by a range from 0 to 100 in score, with a higher score indicating a better QOL. The accuracy of SF-36 using within Chinese HD patients has been confirmed previously (Cronbach’s alpha was 0.9116) [20].

#### Assessment of social support

Perceived Social Support Scale (PSSS) was used to assess social support status. PSSS, containing 12 self-evaluating clauses, can be categorized into family support and outside family support. Each clause is scored from 1 (strongly disagree) to 7 (strongly agree), and higher score indicates a higher social support got by the participant. The reliability and validity of the Chinese Version of PSSS are satisfactory (Cronbach’s alpha was 0.8792) [21, 22].

#### Assessment of coping style

Coping style was assessed by using the Chinese edition of Medical Coping Modes Questionnaire (MCMQ) [23]. It consists of 20 items, including three critical subscales: confrontation, avoidance, and acceptance-resignation. (Cronbach’s alpha was 0.7268, 0.6345, and 0.6486, respectively).

#### Assessment of self-efficacy

Self-efficacy was assessed by using the adapted General Self-efficacy Scale, which was evolved from the English version. It includes 10 items, and each item is scored on a 4-point scale, ranging from strong disagreement (1) to strong agreement (4), and higher score indicates a higher level of self-efficacy. The reliability of the scale is high (Cronbach’s alpha was 0.9174).

#### Assessment of ego resiliency

Ego Resiliency Scale was used to assess ego resiliency [24]. It includes 14 items rated on a 4-point scale, ranging from low level (1) to high level (4), and higher score indicates a higher level of ego resiliency [25, 26]. (Cronbach’s alpha was 0.7868).

### Statistical analysis

Continuous data are presented as means ± standard deviations. Student’s *t*-test was used to compare the means of normally distributed variables between “low mood” and “normal mood”, and the Mann–Whitney U test was used for variables that were not normally distributed. Differences among categorical variables were analyzed by using chi-square test or two-tailed Fisher’s exact test. Factors associated with depressive symptoms were determined by multivariate logistic regression analysis. The variables selected into the multivariable logistic model were the ones which were statistically significant, or had significant impacts on the result according to literature reports.

The Statistical Package for Social Sciences Version 11.5 (SPSS Inc., Chicago, IL, USA) was applied for statistical analysis, and the statistical significance was set at *P* < 0.05 (two- tailed test).

## Results

### Description of demographic and clinical characteristics of patients

Of the 280 patients asked to participate in this study, 40 declined to participate with an 85.7% consent rate, and 13 were excluded for unqualified questionnaire with missing values exceeding 10%. Finally, our study sample was comprised of 227 patients (female, 46.7%; mean age, 49.3 ± 14.3 years, ranging from 17 to 85 years; duration of HD, mean 40 months, ranging from 1 to 103 months). Table 1 shows detailed data.

**Table 1:** Demographic characteristics of HD patients with low and normal mood

	Number of subjects *n* = 227	Normal mood (CES-D < 16) *n* = 161	Low mood (CES-D > =16) *n* = 66	*p* value
Gender, n (%)				0.058
Male	121(53.3%)	85(52.8%)	36(54.6%)	
Female	106(46.7%)	76(47.2%)	30(45.4%)	
Age (years)(mean ± SD)^a^		49.13 ± 14.20	49.65 ± 14.60	0.804
HD duration (months)				0.351
≤ 24	71(31.2%)	54(33.5%)	17(25.8%)	
25–60	78(34.4%)	56(34.8%)	22(33.3%)	
>60	78(34.4%)	51(31.7%)	27(40.9%)	
Marital status, n (%)				0.820
Married	181(79.7%)	129(80.1%)	52(78.8%)	
others	46(20.3%)	32(19.9%)	14(21.2%)	
Educational level				0.922
≤ Junior high school	94(41.4%)	67(41.6%)	27(40.9%)	
>Junior high school	133(58.6%)	94(58.4%)	39(59.1%)	
Monthly income per person (Yuan)				0.008**
<800	42(18.5%)	25(15.5%)	17(25.8%)	
800–1499	49(21.6%)	28(17.4%)	21(31.8%)	
1500–2999	75(33.0%)	58(36.0%)	17(25.8%)	
3000–4999	39(17.2%)	34(21.1%)	5(7.6%)	
≥ 5000	22(9.7%)	16(9.9%)	6(9.1%)	
Primary kidney disease				0.445
Chronic glomerulonephritis	116(51.1%)	87(54.0%)	29(43.9%)	
Diabetic nephropathy	19(8.4%)	13(8.1%)	6(9.1%)	
Hypertension	22(9.7%)	13(8.1%)	9(13.6%)	
Others	70(30.8%)	48(29.8%)	22(33.3%)	
Ca (mmol/L)(mean ± SD)^a^		2.11 ± 0.30	2.18 ± 0.28	0.074
P (mmol/L)(median(IQR))^b,c^		2.08(1.05)	1.95(1.30)	0.768
PTH (pmol/L)(median(IQR))^b,c^		36.67(122.05)	29.12(36.06)	0.300
Total weekly urea Kt/V (mean ± SD)^a^	1.4 ± 0.5	1.4 ± 0.5	1.4 ± 0.5	0.606
Religion				0.850
No	198(87.2%)	140(87.0%)	58(87.9%)	
Yes	29(12.8%)	21(13.0%)	8(12.1%)	
BMI				0.155
<25	182(80.2%)	125(77.6%)	57(86.4%)	
≥ 25	45(19.8%)	36(22.4%)	9(13.6%)	
Concern about arteriovenous fistula				0.032*
Always	67(29.5%)	43(26.7%)	24(36.4%)	
Frequently	37(16.3%)	22(13.7%)	15(22.7%)	
Not at all	123(54.2%)	96(59.6%)	27(40.9%)	
Renal disease duration (months)				0.382
≤ 40	71(31.3%)	46(28.6%)	25(37.9%)	
41–89	82(36.1%)	61(37.9%)	21(31.8%)	
≥ 90	74(32.6%)	54(33.5%)	20(30.3%)	
Activities of daily living		15.4 ± 3.9	18.1 ± 6.3	<0.001***
SF-36 (mean ± SD)^a^				
Physical functioning		81.1 ± 18.3	67.7 ± 24.4	<0.001***
Role limitation due to physical problems		53.1 ± 40.7	28.8 ± 38.0	<0.001***
Role limitation due to emotional problems		78.9 ± 32.6	44.9 ± 45.5	<0.001***
Social functioning		69.4 ± 22.2	47.3 ± 24.8	<0.001***
Mental health		85.7 ± 12.8	54.9 ± 22.9	<0.001***
Energy and vitality		69.1 ± 18.7	42.8 ± 21.0	<0.001***
Bodily pain		82.4 ± 25.7	63.6 ± 34.1	<0.001***
General perception of health		49.3 ± 20.2	29.1 ± 16.9	<0.001***
Social support (mean ± SD)^a^				
Family support		25.3 ± 2.6	23.5 ± 3.6	<0.001***
Outside family support		44.5 ± 8.9	37.7 ± 11.1	<0.001***
Coping style (mean ± SD)^a^				
Confrontation		18.8 ± 4.5	17.9 ± 4.5	0.149
Avoidance		18.1 ± 3.3	17.1 ± 3.1	0.041*
Acceptance-resignation		10.6 ± 1.9	11.9 ± 2.3	<0.001***
Self-efficacy (mean ± SD)^a^		29.7 ± 6.7	24.4 ± 7.6	<0.001***
Ego resiliency (mean ± SD)^a^		41.1 ± 7.9	36.4 ± 6.9	<0.001***

* *p* < 0.05; ** *p* < 0.01; *** *p* < 0.001

^a^ Independent-samples t test

^b^ Median and inter-quartile range

^c^ Mann-Whitney U test

Other causes of ESRD not specified in Table 1 included chronic interstitial nephritis (8.8%), polycystic kidney disease (4.8%), renal allograft loss (3.5%), lupus nephritis (2.2%), purpura nephritis (1.8%), HBV associated glomerulonephritis (1.8%), gouty nephropathy (1.3%), systemic vasculitis (0.9%), obstructive nephropathy (0.4%), trauma (0.4%), and unknown causes (4.9 %).

### Description of the patients’ depressive symptoms

In our study, sixty-six patients (29.1%) suffered from depressive symptoms. Table 1 shows the demographic characteristics of all participants. There was no significant difference between groups of low and normal mood in HD duration, marital status, educational level, religion, primary kidney disease or renal disease duration. Patients with depressive symptoms had lower monthly income and concerned more about arteriovenous fistula.

Table 1 also shows the scores of ADL, QOL, social support, coping style, self-efficacy and ego resiliency. Patients with a lower mood have worse ADL and QOL than patients with better mood. For both family support and outside family support, patients with a lower mood have got less social support than patients with better mood. Patients with a lower mood more inclined to choose “acceptance-resignation” coping style than patients with better mood, while the result is opposite in “avoidance” coping style. And patients with a better mood have better self-efficacy and ego resiliency than patients with lower mood.

### Multivariate logistic regression analysis for related factors

Multivariate logistic regression analyses revealed that ADL (OR = 1.124, *p* = 0.002), family support (OR = 0.867, *p* = 0.021), “acceptance-resignation” coping style (OR = 1.228, *p* = 0.022), and ego resiliency (OR = 0.944, *p* = 0.021) were independently associated with low mood (Table 2).

**Table 2:** The multivariate logistic regression analysis for exploring factors of depressive symptoms

Variables	OR	OR 95%CI	*p* value
Age	0.990	0.963 ~ 1.017	0.457
Gender (male vs. female)	0.798	0.379 ~ 1.680	0.552
Activities of daily living	1.124	1.043 ~ 1.212	0.002**
Social support			
Family support	0.867	0.768 ~ 0.979	0.021*
Outside family support	0.972	0.935 ~ 1.010	0.148
Coping style			
Confrontation	0.980	0.903 ~ 1.062	0.619
Aviodance	0.966	0.861 ~ 1.084	0.558
Acceptance-resignation	1.228	1.030 ~ 1.466	0.022*
Self-efficacy	0.965	0.914 ~ 1.019	0.202
Ego resiliency	0.944	0.899 ~ 0.991	0.021*
HD duration (months) (>60 vs. ≤24)	2.154	0.860 ~ 5.395	0.101
HD duration (months) (25–60 vs. ≤24)	1.182	0.486 ~ 2.878	0.712
*p < 0.05; ** *p* < 0.01			

## Discussion

In our study, the prevalence of depressive symptoms in CKD patients on HD was 29.1%. The result is consistent with previous data showing a prevalence of depression in patients on HD ranges from 23% to 45.9% [7, 8]. However, the result is a little higher than the prevalence of depression among pre-dialysis CKD patients [27, 28]. Compared with the population in developed countries, Chinese often feel embarrassed to seek mental health care because of the cultural issues, so that the mental health status of Chinese has been ignored for decades, and there are less studies focus on mental health of patients on HD [29, 30]. Therefore, summarizing the prevalence of depressive symptoms in patients on HD in northern China is an important first step, then developing additional research priorities [31].

Patients with a lower mood have worse ADL than patients with better mood. Also, ADL was associated with low mood independently. Our finding was in agreement with some previous studies [32]. Patients in ESRD struggled to carry out normal daily life despite the dependency caused by the illness [33]. The reality that they could not finish routine activities independently, such as bathing or doing housework may be associated with depressive symptoms. In a research among 5763 patients on HD, the findings showed that aerobic physical activity was associated with fewer depression symptoms [34]. Finding some effective physical activity regimens which were suitable for Chinese HD people to improve the functional status may be a feasible scheme to reduce depressive symptoms in patients under HD treatment.

In our study, we found that patients with a lower mood received less social support than patients with better mood, including both family support and outside family support. And family support was negatively associated with low mood. Our finding was in agreement with previous study in patients on peritoneal dialysis (PD) in southern China [35]. Social support refers to the perception and actuality that one gets assistance from other people [36]. HD as a long-term management strategy, patients under HD treatment may lose personal freedom and social connectedness [37]. The characteristic of HD treatment changes the life style of family caregivers too. It is important that not only for the patients, but also for the family members to focus on preparation for the change to the new way of life [38]. According to our result, family support is associated with low mood, the support received from family is more important to the patients under HD treatment. For example, patients relied on spouses and siblings for help with transportation to and from the dialysis unit, and discussed with them the difficulties associated with HD [39, 40]. As family support is highly valued, the family members should give more advice and help.

In assessing the coping style, patients with a lower mood more like to select “acceptance-resignation” coping style than patients with better mood. And “acceptance-resignation” coping style was associated with low mood independently. This result was in agreement with previous study in patients on peritoneal dialysis (PD) in southern China [35]. Patients on HD must confront with constant threat of diseases and death, which will impose them many challenges and difficulties, and will often require new and different ways to cope with. This result suggested that patients with low mood were inclined to the usage of “acceptance-resignation” coping style. Use of this type of coping style indicates a lack of hope, shows that patients under HD regard their disease as non-solving, so it is not surprising that “acceptance-resignation” coping style are associated with low mood in patients on HD.

In our study, we also found that patients with a better mood have better ego resiliency than patients with lower mood. And ego resiliency was independently associated with low mood. Ego resiliency is an individual’s ability to adapt to stress and adversity. Individuals with better ego-resiliency are likely to adjust well to changing environment [41]. Thus, physicians should evaluate ego-resiliency of HD patients, and help them developing ego-resiliency may moderate the happening of the depressive symptoms.

We could speculate on the situation of the whole population according to results of this study, based on the property of cluster sampling. However, several limitations of our study should be considered. First, patients recruited in our study were only from two affiliated hospitals which may limit to public hospitals in city. Second, since this study is a cross-sectional study, we couldn’t get causality based on our findings, further longitudinal studies with treatment interventions is necessary.

## Conclusions

In conclusion, based on cross-sectional survey, our findings revealed that in HD patients in northern China, the prevalence of depressive symptoms was high. ADL, family support, “acceptance-resignation” coping style and ego resiliency were independently associated with depressive symptoms. Physicians should give more attention to prevent and treat depressive symptoms among HD patients.
